# Effectiveness of Postoperative Radiotherapy on Atypical Meningioma Patients: A Population-Based Study

**DOI:** 10.3389/fonc.2019.00034

**Published:** 2019-01-31

**Authors:** Qiang Zeng, Feina Shi, Zhige Guo

**Affiliations:** ^1^Department of Neurosurgery, Second Affiliated Hospital of Zhejiang University School of Medicine, Hangzhou, China; ^2^Department of Neurology, Second Affiliated Hospital of Zhejiang University School of Medicine, Hangzhou, China

**Keywords:** atypical meningioma, radiotherapy, prognosis, surgery, gross-total resection, subtotal resection

## Abstract

**Purpose:** It is controversial whether atypical meningioma patients undergoing gross-total resection (GTR) can benefit from postoperative radiotherapy (PORT). This study aimed to investigate the effectiveness of PORT on atypical meningioma patients.

**Methods:** Patients diagnosed with atypical meningioma from 2008 to 2015 were extracted from the Surveillance, Epidemiology, and End Results (SEER) database. The Kaplan–Meier survival curves were generated, and the log-rank test was used to compare the differences among groups. Univariable and multivariable COX regressions were conducted for survival analyses.

**Results:** A total of 1,014 patients were enrolled. The 5-years survival rate of the overall patients was 79.0%. PORT was performed in 315 (31.1%) patients. The utilization rates of PORT in patients undergoing GTR and undergoing subtotal resection (STR) were 26.7% and 42.2%, respectively. For patients undergoing STR, log-rank test showed that overall survival (OS) time was significantly longer in patients receiving PORT than those not (*p* = 0.026). For patients undergoing GTR, OS time did not show significant association with PORT (*p* = 0.339). In addition, patients undergoing STR with PORT had no significantly different OS time compared with those undergoing GTR with PORT (*p* = 0.398). Multivariable Cox regression analysis showed that receipt of PORT (*p* = 0.187) was not an independent predictor of OS after adjustment.

**Conclusion:** PORT may not prolong the OS in atypical meningioma patients undergoing GTR. However, patients undergoing STR may benefit from PORT and achieve similar OS to those undergoing GTR.

## Introduction

Meningiomas are the most common primary intracranial tumors with an incidence rate of about 8 per 100,000 population, accounting for ~37% of all central nervous system tumors (1). According to WHO 2016 classification, it can be divided into WHO grades I–III (2), of which more than 80% cases are classified as WHO grade I, with 4–15% cases classified as WHO grade II (atypical meningioma) and 1–3% cases classified as WHO grade III (anaplastic meningioma) (3, 4).

Postoperative radiotherapy (PORT) is routinely recommended for meningioma patients undergoing subtotal resection (STR), while for those undergoing gross-total resection (GTR), postoperative therapeutic strategies vary depending on grades (3, 5). After GTR, observation is recommended for patients with WHO grade I meningioma, and PORT is suggested for patients with anaplastic meningioma (3, 5). However, optimal management for atypical meningioma patients after GTR is still controversial, and it is contentiously debated whether these patients can benefit from PORT. Several small studies have been performed to investigate the effect of PORT on atypical meningioma patients after GTR, but led to contradictory results (6–15). A phase II clinical trial (RTOG 0539) suggested that PORT can improve 3-year progression-free survival for intermediate-risk meningioma patients (16).

A recent study based on the National Cancer Database found that GTR and PORT were associated with improved survival for patients with atypical meningioma (17). However, patients who died within 1 month after surgery, most of whom had no opportunity to receive postoperative radiotherapy, were not excluded in that study, which might lead to an abrupt drop at the beginning of the Kaplan–Meier survival curve of patients not receiving PORT, resulting in a false positive result in the comparison of overall survival (OS) between patients receiving and not receiving PORT. Therefore, we aimed to perform this population-based study to assess the effect of PORT on survival outcomes in atypical meningioma patients after excluding those who had an OS time <1 month to reduce the selection bias.

## Materials and Methods

### Patients

**Figure 1:**
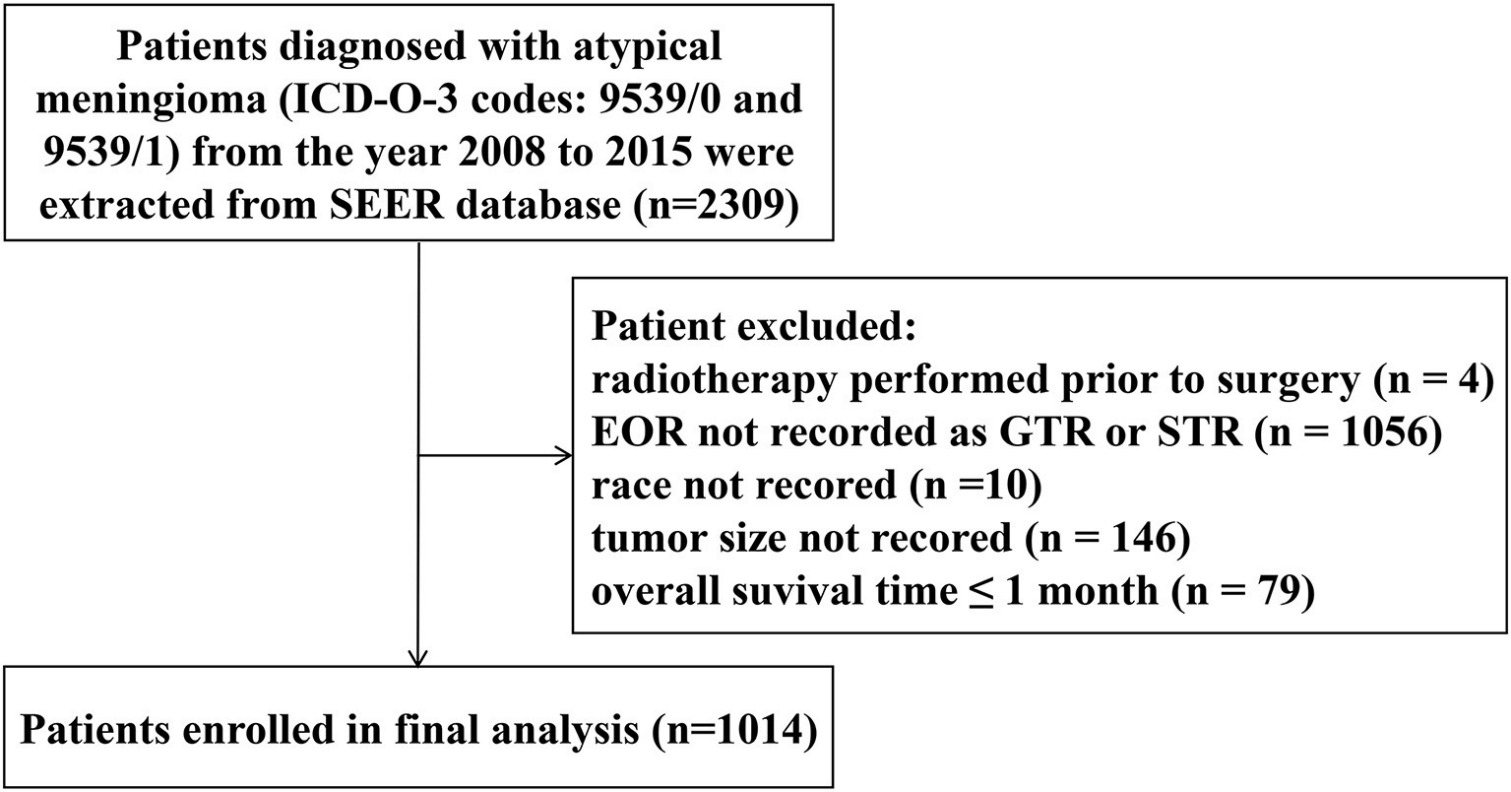
Flow diagram of patient selection. EOR, extent of resection; GTR, gross-total resection; STR, subtotal resection.

This study was performed based on the Surveillance, Epidemiology, and End Results (SEER) database (18). Patients diagnosed with atypical meningioma from the year 2008 to 2015 were identified using the SEER^*^Stat software (Version 8.3.5), with *International Classification of Diseases for Oncology, Third Edition* (ICD-O-3) codes 9539/0 and 9539/1. The demographic and clinical data of the patients were extracted from the database, including age at diagnosis, gender, race, tumor size, extent of resection (EOR), radiation sequence with surgery, survival time and vital status. Patients were excluded if (1) radiotherapy was performed prior to surgery; (2) EOR was not recorded as GTR or STR; (3) race was not recorded; (4) tumor size was not recorded; (5) OS time ≤1 month. The flow diagram of patient selection is shown in Figure 1. This study was ruled exempt from review by the ethics review board at the Second Affiliated Hospital of Zhejiang University school of Medicine.

According to the coding instructions of the SEER database (SEER Program Coding and Staging Manual 2018 (draft), available at: seer.cancer.gov/tools/codingmanuals), PORT here refers to first-line postoperative adjuvant therapy, and radiotherapy performed after 1 year of diagnosis or after relapse was not counted. Generally, for meningioma, GTR represents a Simpson grade I, II, or III resection, and STR represents a Simpson grade IV or V resection (19). However, the detailed associations between Simpson grade and GTR or STR were not available from the SEER database.

### Statistical Analysis

All statistical analyses were performed with SPSS statistics, v. 22 (IBM, Armonk, NY). Medians with interquartile ranges (IQR) and percentages were used to describe the distribution of continuous and categorical variables, respectively. The Chi-Square test was used to compare differences between two groups for categorical variables. The Kaplan–Meier survival curves were generated, and the log-rank test was used to compare among groups. Univariable and multivariable COX regressions were conducted for survival analyses. Variables with a *p* < 0.20 in univariable analyses were included in multivariable analysis. A *p* < 0.05 was considered statistically significant.

## Results

### Patient Characteristics

A total of 1,014 patients were enrolled in the final analysis. The demographic and clinical characteristics of patients were summarized in Table 1. The median age of the patients was 60 years (IQR, 48–69 years). There were 567 (55.9%) females. GTR was achieved in 727 (71.7%) patients.

**Table 1:** Demographic and clinical characteristics of patients with atypical meningioma.

**Characteristic**	**Value**
Age, years	60 (48–69)
Female, *n* (%)	567 (55.9%)
**Race**	
Black, *n* (%)	152 (15.0%)
White, *n* (%)	744 (73.4%)
Other, *n* (%)	118 (11.6%)
Tumor size, cm	4.9 (3.7–6.0)
GTR, *n* (%)	727 (71.7%)
PORT, *n* (%)	315 (31.1%)

*GTR, gross-total resection; PORT, postoperative radiotherapy*.

PORT was performed in 315 (31.1%) patients. The utilization rates of PORT in the patients undergoing GTR and undergoing STR were 26.7% (*n* = 194) and 42.2% (*n* = 121), respectively. For the patients undergoing STR, the utilization rate was significantly lower in the elder patients (age ≥ 60 years) than in the young patients (age < 60 years) (49.3 vs. 35.4%, *p* = 0.017).

### Survival Analysis

**Figure 2:**
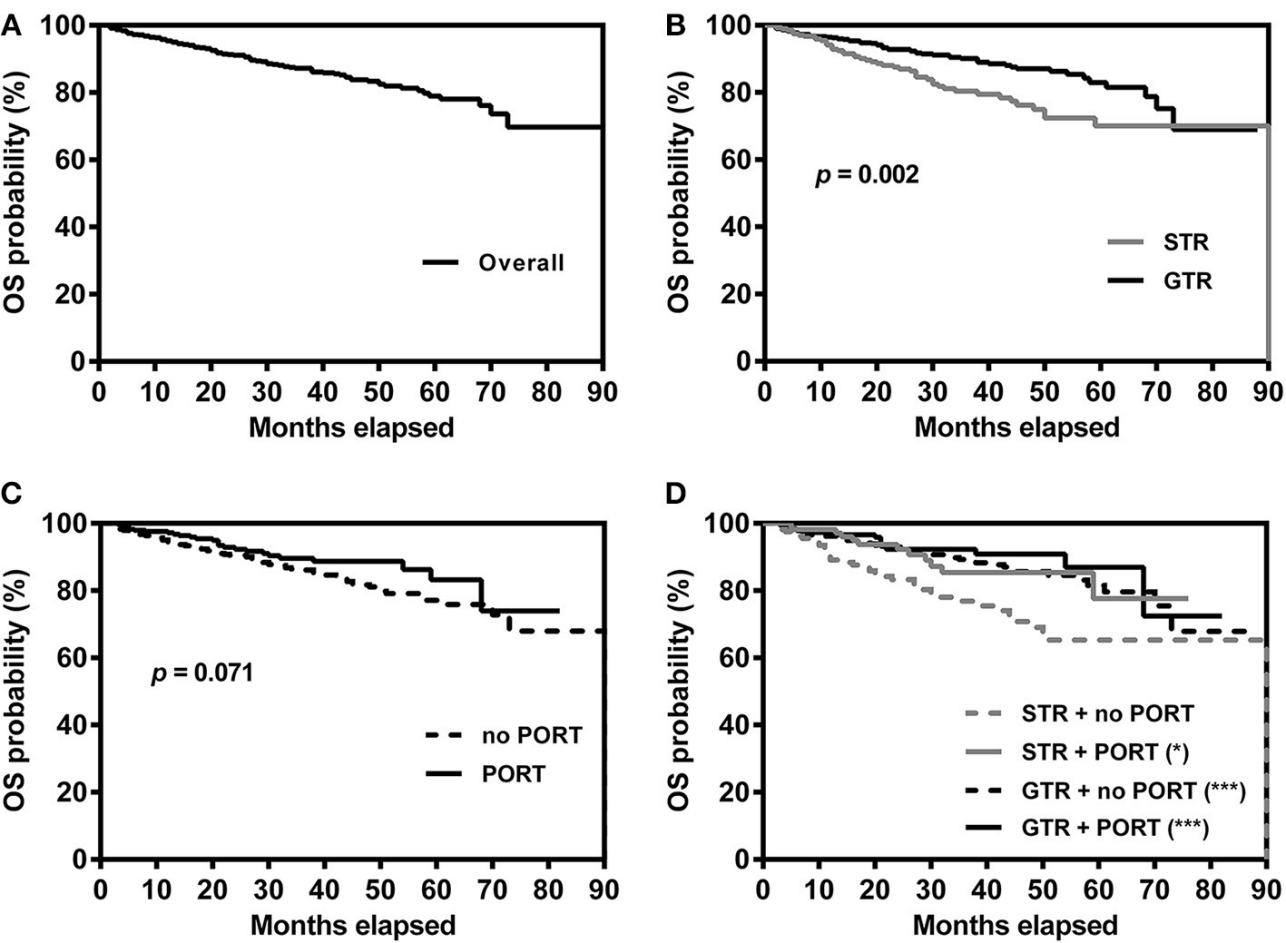
Kaplan–Meier survival curves for overall patients **(A)**, patients grouped by extent of resection **(B)**, patients grouped by receipt of PORT **(C)** and patients grouped by extent of resection and receipt of PORT **(D)**. ^*^p = 0.026, ^***^p < 0.001, compared with patients undergoing STR without PORT. GTR, gross-total resection; STR, subtotal resection; PORT, postoperative radiotherapy.

The Kaplan-Meier survival curves are shown in Figure 2. The 1-, 3-, and 5-years survival rates of the overall patients were 95.3, 87.2, and 79.0%, respectively. The survival rates of the patients grouped by EOR and receipt of PORT was listed in Table 2. For the patients undergoing STR, OS time was significantly longer in the patients receiving PORT than those not (*p* = 0.026). For the patients undergoing GTR, there were no significant difference in OS time between the patients receiving and not receiving PORT (*p* = 0.339). In addition, the patients undergoing STR with PORT had no significantly different OS time with the patients undergoing GTR with PORT (*p* = 0.398).

**Table 2:** Survival rates of the patients grouped by extent of resection and receipt of postoperative radiotherapy.

**Group**	**1-year survival rate (%)**	**3-years survival rate (%)**	**5-years survival rate (%)**
STR	92.9	80.3	70.0
GTR	96.3	90.1	83.0
No RT	94.5	86.1	77.1
RT	97.2	89.6	83.2
GTR without PORT	96.2	89.3	81.5
GTR with PORT	96.7	92.3	86.9
STR without PORT	89.1	76.9	65.3
STR with PORT	98.2	85.4	77.6

*GTR, gross-total resection; STR, subtotal resection; PORT, postoperative radiotherapy*.

Univariable Cox regression analysis showed that GTR was associated with improved OS (HR [95% CI] = 0.56 [0.38–0.81], *p* = 0.002) and older age was associated with poor OS (HR [95% CI] = 1.07 [1.06–1.09], *p* < 0.001), shown in Table 3. Tumor size and receipt of PORT tended to affect OS (*p* = 0.162 and 0.074, respectively). Gender and race were not associated with OS (*p* = 0.661 and 0.319, respectively). The multivariable Cox regression analysis showed that receipt of PORT (*p* = 0.187) was not an independent predictor of OS after adjustment. However, EOR (HR [95% CI] = 0.51 [0.35–0.75], *p* < 0.001) could independently predict OS after adjustment.

**Table 3:** Univariable and multivariable Cox model of variables in predicting overall survival in patients with atypical meningioma.

**Variable**	**Univariable analysis**			**Multivariable analysis**		
	**HR**	**95% CI**	***p*-Value**	**HR**	**95% CI**	***p*-Value**
Age	1.07	1.06–1.09	<0.001	1.07	1.06–1.09	<0.001
Gender (Female vs. Male)	1.09	0.75–1.57	0.661			
**Race**			0.319			
White vs. Black	0.70	0.44–1.11	0.136			
Other vs. Black	0.71	0.36–1.40	0.321			
Tumor size	1.01	1.00–1.02	0.162	1.01	1.00–1.02	0.189
EOR (GTR vs. STR)	0.56	0.38–0.81	0.002	0.51	0.35–0.75	<0.001
PORT vs. not	0.67	0.43–1.04	0.074	0.74	0.47–1.16	0.187

*EOR, extent of resection; GTR, gross-total resection; STR, subtotal resection; PORT, postoperative radiotherapy*.

## Discussion

Optimal postoperative management for atypical meningioma patients undergoing GTR remains a great deal of controversy. It is unclear whether these patients can benefit from PORT. By using the SEER database, the current study found that PORT could not prolong the OS time in atypical meningioma patients undergoing GTR, which indicates that these patients may not benefit from PORT after GTR. Several previous studies have attempted to address the controversy about whether PORT can improve the outcome in atypical meningioma patients undergoing GTR. Some studies did not show significant survival benefit from PORT in these patients (8, 10–14). However, the missing significance has been suspected as a result of small sample size, and some studies also found a contradictory result that PORT could improve the outcome (6, 7, 9, 15). Thus, studies with large sample size are needed to settle the dispute. A recent meta-analysis, with a total of 757 patients included, showed that PORT decreased the risk of tumor recurrence but did not affect the survival time because most recurrent tumors were salvageable with surgery or radiation (20). A previous study based on the SEER database performed by Stessin's et al. found that PORT did not impart a survival benefit for the patients with grade II and III meningioma after adjustment (21). However, that study enrolled patients diagnosed with meningioma between 1988 and 2007. Brain invasion has been added as a grading criterion for grade II meningioma in the 2007 WHO classification and new 2016 WHO classification, and the proportion of WHO grade II meningioma has obviously increased since 2007 (16). By enrolling 1,014 patients diagnosed with atypical meningioma between 2008 and 2015, our study provides further evidence that PORT may not prolong the survival time in patients undergoing GTR.

The reported incidence of treatment toxicity for atypical meningiomas after PORT ranged from 3.4 to 16.7% (22). Although side effect of radiotherapy is usually mild, it has been pointed out that radiotherapy may increase the risk of malignant transformation (3). Besides, blindness occurred in about 5% of patients receiving 50 Gy radiation due to irradiation of the optic apparatus, and seizure was reported in 4.2% of patients (22, 23).

Nevertheless, in accordance with previous studies (11, 15), our study showed a significant survival benefit from PORT for patients undergoing STR, indicating that PORT should be performed for atypical meningioma patients undergoing STR. Although PORT has been routinely recommended for atypical meningioma patients undergoing STR (3, 5), it is surprising that the utilization rate of PORT was only 42.6% in these patients and even lower in the elder patients. These results indicate that atypical meningioma patients undergoing STR receive insufficient postoperative treatment and that adherence to clinical guidelines need to be improved for these patients, especially for elder patients.

It is well-estimated that EOR is an important predictor of prognosis in atypical meningioma patients (10, 13–15). The current study also found that patients undergoing GTR had significantly longer OS compared with those undergoing STR, and EOR was an independent predictor of OS after adjustment. However, it is interesting to find that the patients receiving PORT after STR could achieve a similar OS with the patients receiving PORT after GTR. Thus, EOR may not affect the OS if PORT is routinely performed for patients undergoing STR. However, PORT may be unnecessary for patients undergoing GTR as PORT may not be able to prolong the OS. Thus, patients can also benefit from GTR, and GTR should be attempted when patients undergo surgery.

Perioperative mortality was typically defined as death within 1 month after surgery (24, 25). The reported perioperative mortality rate of meningioma patients was 1.9 or 6.1% (25, 26). Most of the patients who die within 1 month after surgery do not have the opportunity to receive postoperative radiotherapy. Including these patients in analyses will lead to an abrupt drop at the beginning of the Kaplan–Meier survival curve of patients not receiving PORT, which can be seen in several previous studies (4, 11, 17). This may result in a false positive result in the comparison of OS between patients receiving and not receiving PORT. Thus, we recommend that patients with very short survival time should be excluded in such studies. In the current study, we excluded patients who had an OS time ≤1 month to reduce the selection bias.

The major strength of this study is the large sample size. However, there are several limitations in this study. First, this is a retrospective study, and inherent limitation exists in this kind of study, such as potential selection bias in treatment modalities offered. Second, progression free survival (PFS) time is not available from the SEER database, making it impossible to analyze the effect of PORT on PFS in atypical meningioma patients. Third, some other important information, such as Simpson grade and status of brain invasion, was also not available from the SEER database. Further heterogeneity in overall survival may exist when stratified by the status of brain invasion or Simpson grade. Forth, the PORT techniques performed on the patients were not standardized. Fifth, the record of PORT in the SEER database is not completely certain. PORT received outside of the hospital setting might be not captured in the SEER, then the patient might be misclassified into no PORT group. Considering such limitations in this study, a well-designed multicenter prospective study is needed to further investigate the impact of PORT on the outcome of atypical meningioma patients. Currently, two phase III trials, ROAM-EORTC 1308 (27) and NRG-BN003 (https://clinicaltrials.gov/ct2/show/NCT03180268), are now ongoing to evaluate the effectiveness of adjuvant radiotherapy in reduction of recurrence risk in atypical meningioma patients who have undergone gross total resection. The results of these well-designed trials will help to address the controversy about the effectiveness of PORT in atypical meningioma patients.

In conclusion, PORT may not prolong the OS in atypical meningioma patients undergoing GTR. However, patients undergoing STR may benefit from PORT and achieve similar OS to patients undergoing GTR. Although PORT has been routinely recommended for patients undergoing STR, the utilization rate of PORT was less than half in these patients. The results suggest that PORT should be performed in atypical meningioma patients undergoing STR and may not be recommended for those undergoing GTR. Further prospective studies are needed to identify the impact of PORT on atypical meningioma patients after GTR.

## Author Contributions

QZ conceived the idea. QZ and ZG collected the data. QZ and FS analyzed the data. QZ drafted the manuscript. All authors reviewed the manuscript.

### Conflict of Interest Statement

The authors declare that the research was conducted in the absence of any commercial or financial relationships that could be construed as a potential conflict of interest.
